# “The birth of the association of European National Olympic Committees and its stakes during the 1960s to 1970s”

**DOI:** 10.3389/fspor.2025.1654715

**Published:** 2025-10-08

**Authors:** Florent Lefèvre

**Affiliations:** Performance, Health, Metrology, Society Laboratory (EA 7507) of the Training and Research Unit (UFR) for Physical and Sports Activities Sciences and Techniques (STAPS), University of Reims Champagne Ardenne, Reims, France

**Keywords:** olympism, IOC, AENOC, history, Olympic games

## Abstract

The initiative to associate the European National Olympic Committees was launched in the 1960s by the French Olympic Committee and its president at the time, Count Jean de Beaumont. This project faced hesitations and difficulties when it came to realization. The International Olympic Committee, led by Avery Brundage, notably obstructed it, as he was concerned about not disrupting the overall governance of the Olympic Movement. These years marked the beginning of certain alliances, particularly European ones, within the IOC in response to Brundage’s presidency. Indeed, for the IOC president, the initiatives of Onesti and Beaumont were aimed at targeting the presidency of the IOC. It was during this period that new institutional alliances emerged, with networks of men and women finding themselves at the heart of complex relational knots, continually hindered by opposing institutional forces and constantly energized by unique personal encounters. This highlights the emergence of new institutions, ideologies, and visions of Olympism that developed within the Olympic Movement and around the IOC, and sometimes against it. President Brundage viewed these initiatives from the National Olympic Committees as attempts to undermine the authority of the IOC. According to him, it is the IOC that represents and brings together all the NOCs, not the Permanent General Assembly of the NOCs or the General Assembly of the NOCs of Europe. To promote these issues and alliances, numerous original IOC archives were studied. This research therefore aims to highlight the issues surrounding the creation of the Association of European NOCs while focusing on the Olympic context of the 1960s.

## 1 Introduction

**Figure 1:**
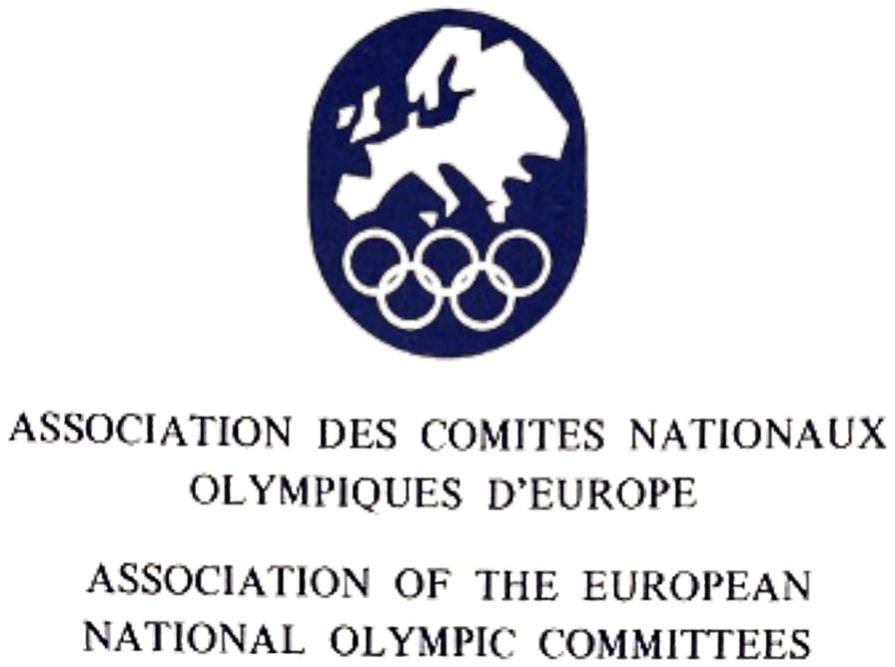
AENOC statutes in 1975. Source: First statutes of the AENOC, 1975, Archives of the French National Olympic and Sports Committee.

As shown by B. Lepetit's works, analysing the role of the various actors to better understand the different alliances set up around the creation of the Association of European National Olympic Committees (AENOC) during the 1960s allows us to highlight the acts and actions of each one within a collective system (Figure 1) (1). The aim of this article is to explain the nature of the alliances and opposition that led to the slow and controversial institutionalisation of the AENOC during the 1960s. Based on a main corpus of correspondence between the various actors, the article intends to bring to light the power struggles between successive AENOC advocates and permanent opponents, such as IOC President Avery Brundage and representatives of the British NOCs. In other words, the aim is to show how the correspondence between these actors allows us to understand in what way and why the personal stakes of some actors, seeking mainly to increase their responsibilities within Olympic governance, developed and interfered with the geopolitical issues asserting themselves during the 1960s and linked to the evolution of international relations within European space and of European political governance.

Specific attention was therefore paid to analysing the challenges surrounding the creation of the Association of European NOCs and its links with the IOC, in order to better understand the reality of European Olympic unity in the 1960s and 1970s. Within this dynamic, the IOC's position regarding the creation of the AENOC, and more broadly the Association of NOCs (ANOC),^1^ was of paramount importance.

**Figure 2:**
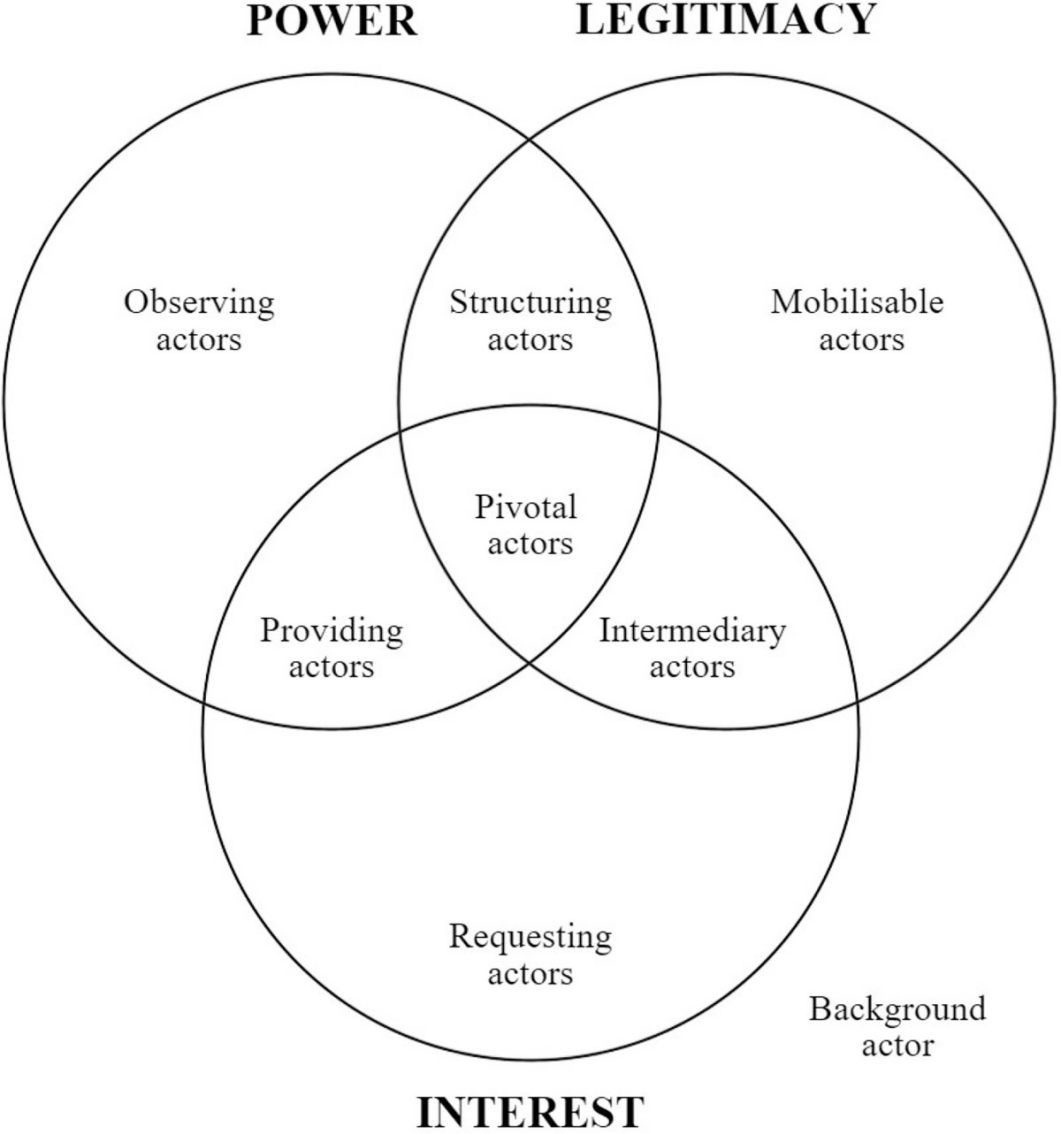
The typology of actors according to their attributes.

While the dynamics between actors explains the controversial institutionalisation process of the ANOC, followed by that of the AENOC, analysing European Olympic governance during the 1960s and 1970s remains “essential to understanding the conditions of implementation and sustainability of these initiatives” (2). The typology of actors, used by Brullot, Maillefert and Joubert in 2014 makes it possible to better characterise these actors according to their position and involvement by placing them as for or against the NOCs' new forms of governance, while simultaneously revealing actor power, legitimacy, and value (3) (Figure 2).

In terms of the corpus used, this analysis is based on an in-depth study of the archives housed at the IOC's Olympic Studies Centre in Lausanne, with priority given to analysing the correspondence. Several archive collections were thus examined. Firstly, the collections of the two institutions concerned: the Association of European National Olympic Committees (AENOC) and the Association of National Olympic Committees (ANOC), both institutions being closely linked by their actors, goals, and challenges. In addition, and with the aim of identifying the actions of individuals, a number of archive collections regarding individual members (IOC members and non-members) directly involved in the creation history of the AENOC were also examined. In order to cross-check the data regarding European and international Olympism, the archive collections of Avery Brundage and Lord Killanin were likewise studied. Lastly, documents concerning the founding fathers of the ANOC and the AENOC were examined, on the one hand, IOC members, such as Count Jean de Beaumont (France), Giulio Onesti (Italy), Raymond Gafner (Switzerland), Willi Daume (Germany), and on the other, non-members of the IOC, such as the Swiss Jean Weymann,^2^ Frenchmen Alain Danet^3^ and Claude Collard,^4^ and the Belgian Raoul Mollet.^5^ Cross-checking sources in this way made it possible to understand the diversity of the points of view expressed, and assess both the extent of the IOC's control over its members and the reaction of non-members.

Among this group of institutional actors, precisely identified within the correspondence, four pivotal actors asserted themselves as the leaders and coordinators of AENOC; Giulio Onesti, Count Jean de Beaumont, Raoul Mollet and Raymond Gafner represented the “four musketeers” of this endeavour (2). They were moreover joined by Jean Weymann, Luc Silance, and Alain Danet. Despite their generous intentions, they had to face opposition embodied by IOC President Avery Brundage and Ivar Vind (Denmark), General José de Clark (Mexico), as well as the British NOCs.

Ultimately, in addition to the 60 excerpts of private and public correspondence analysed, the corpus was also enriched with documents from the archives of the French National Olympic and Sports Committee (FNOSC), the German Olympic Sports Confederation (GOSC) and the French National Archives.^6^

Regarding the temporality of this research, the period between 1965 and 1975 is usually considered as that of the AENOC's creation. 1965 represents the first international general assembly of worldwide NOCs, held in Rome on the initiative of Giulio Onesti. The endpoint of this study relates to the signature of the first statutes, officially creating the Association of European National Olympic Committees during the general assembly of European NOCs in Lisbon. In the first instance, the aim is to focus on the birth of this global forum of NOCs. The actors involved in uniting NOCs worldwide, as well as their objectives, were analysed. In fact, this first initiative resulted in the need to create a European NOC (ENOC). It was in this context that, three years later, in 1968, European NOCs gathered at Versailles on the initiative of the French NOC and its president Count Jean de Beaumont. The road towards the official creation of the AENOC was thus opened. The first part of this article focuses on an in-depth study of the context within the Olympic Movement (OM) at the turn of the 1960s and, more specifically, on the often challenging relations between a rather conservative IOC and the intention of some NOCs to create an organisation sealing their European identity. The second part of the article investigates the difficult institutionalisation of the AENOC between 1968 and 1975.

About the litterature review, this research work is at the crossroads of numerous studies already carried out on the Olympic Movement. Furthermore, some research has looked at the evolution of Olympism over time, the challenges of change and, more generally, its resilience (Chatziefstathiou 2011), (Durantez 1993), but very little at the crisis between the NOCs and the IOC. The Olympic Games have become a global cultural event (MacAloon, 2008), but it is essential to question the role and history of the NOCs in this Olympic story. Indeed, since the process of decolonisation, the number of NOCs has increased, doubling between the London Games in 1948 and the Munich Games in 1972 (4). On the other hand, some authors, such as Professors Jean-Loup Chappelet and Allen Guttmann, mention in their research the creation of ANOC against Brundage's wishes. The Olympic Movement is marked by an apparent multicultural orientation, but also by a dominant Eurocentrism (DaCosta, 2002). The initiative to bring together all the European NOCs was born out of the crisis in relations between the IOC and the NOCs. This was a period of great tension between, on the one hand, the more progressive Olympic members of the European NOCs and, on the other, the rigid IOC led by the American Brundage.

The NOCs wanted to play a more important role within the Olympic Movement. It was then in 1965, at a meeting in Rome organised by the influential Italian NOC, which had very substantial financial resources at the time (5). Its President, Giulio Onesti, then created the Permanent General Assembly of the NOCs (PGA), against Brundage's wishes (6). It should also be noted that the International Federations did the same in 1967 when they founded the General Assembly of International Sports Federations (GAISF, renamed the General Association of International Sports Federations in 1976), notably under the impetus of Frenchman Roger Coulon (4). The PGA and the GAISF were rivals for the IOC's position in the leadership of world sport, and the latter in particular wanted a new Olympic Congress to be set up (4).

In his historical work published in 2000, Pierre Morath retraces the history of the IOC in Lausanne. At the end of the book, he uses a chronological table to review the key dates in the history of Olympism worldwide. It is within this chronology that he highlights two key events with the foundation of the General Association of International Sports Federations (GAISF) and the NOCs’ PGA. In his words, “this decision was taken in disagreement with Brundage, who considered it unnecessary and who saw in the increased power of the IFs a means of gaining access to national representation within the IOC” (7). Then on the NOCs’ PGA Brundage is opposed to this initiative, ‘which he considers pointless, just as he was opposed to the GAISF initiative’ (8).

In 1994, as part of the celebration of the IOC's centenary, the IOC published the Centenary Book, in three volumes, looking back at the history of the institution, its members, its presidents, the Games and the spread of Olympism. These works are coordinated and directed by Raymonf Gafner. A group of renowned researchers have been selected by President Samaranch to write the centenary history of the IOC. This group is made up of Prof. Dr. Yves-Pierre Boulongne, Prof. Fernand Landry, Prof. Karl Lennartz, Pr. Dr Otto Schantz, Pr. Magdeleine Yerlès and Pr. Dr. Norbert Müller who is the scientific coordinator. In volume II, the part dealing specifically with the presidency of the American Avery Brundage (1952–1972) is produced by Prof. Otto Schanz. In the course of this presentation, the issue of the NOCs’ PGA is raised, as well as the NOCs’ demands in the 1960s more generally. The author focuses on Brundage's criticism of the NOCs’ PGA, which was being built alongside or in opposition to the IOC. It is easy to understand, thanks to archival references, that this initiative caused quite a stir and Brundage did his utmost not to acknowledge it, while trying to ‘bury the PGA’ (9).

Aside from these works that have looked at NOC initiatives on a global scale, none of them mention the initiative to unite the European NOCs. Only two scientific articles have been identified as looking specifically at the Association of National Olympic Committees of Europe. Renaud David and Eric Monnin first published an article in 2011 entitled ‘The Olympic movement and Europe: history and current situation of the Association of European Olympic Committees’. Then in 2012, another publication entitled ‘From Versailles to Brussels: The origins, hesitations and actions of the Association of European Olympic Committees’ was published in connection with the history of AENOC. The two articles by Monnin and David in 2011 and 2012 provide a general understanding of the key moments and issues surrounding the genesis of AENOC.

In order to construct the official and unofficial history of an institution that has been rarely, if ever, studied, the approach taken was to search for archives related to ACNOE. However, the fact that there is little or no research on ACNOE allowed for a certain flexibility in the search for archives, while avoiding the risk of becoming scattered and lost in a very large quantity of collected archives. On the other hand, the methodological approach used aims to highlight a certain desire to cross-reference sources in order to put the different positions and points of view of the actors into perspective. Therefore, the archives consulted at the Olympic Studies Centre in Lausanne were cross-referenced with other archive centres.

## 2 From 1965 to 1968: the time of intentions

### 2.1 The first general assemblies of the NOCs

At the turn of the 1960s, the IOC faced difficult relations with the various NOCs. In fact, numerous NOCs put forward initiatives intended to enhance communication between the IOC and NOCs while serving the Olympic ideal.^7^ The NOCs represent the IOC in their respective territories. The mission of the NOCs is to develop, promote and protect Olympism and its values in their respective countries, in accordance with the Olympic Charter. An NOC also prepares the national delegation to participate in the Olympic Games.

It was initially during the 1964 IOC Session in Tokyo that the IOC-NOC communication issue was raised. In an attempt to confront the problem during the session, Onesti suggested organising “an international conference of NOCs in Rome one year later” (10). Given the increasing number of demands, there was a fear that the NOCs would create a rival organisation (11). As a result, the IOC Executive Board accepted Onesti and the Italian NOC's idea in Tokyo of gathering international NOCs in Rome (12). It was indeed becoming clear that the Olympic Movement was undergoing great change on an international scale. The process of decolonisation had begun and many new NOCs, representing new countries, were created. These emerging NOCs were willing to participate in the international Olympic action, but with no means and hardly any members within the IOC, they felt left out.

The main objective of the Rome General Assembly was to examine how the IOC-NOC relationship could be improved, while supporting the development and action of the NOCs under the authority of the IOC. The first NOC general assembly took place in Rome from 30 September to 2 October 1965 “in the presence of 68 NOCs, of which 27 were from Europe, 15 from Africa, 13 from the two Americas, 12 from Asia, along with the Australian NOC (13). The assembly was inaugurated by IOC President, Avery Brundage, underlining the importance given to the initiative by the IOC.

Juan Antonio Samaranch also took part in this first general assembly of NOCs as Vice President of the Spanish Olympic Committee, and the Spanish NOC was favourable to the idea of an association of NOCs. According to Samaranch, this association would be of great service to sport in general and Olympism in particular, if it was well led. However, Samaranch and his committee did not wish for this meeting to give rise to an NOC association that would rival the IOC. Uniting the NOCs was important, and it was necessary to create a parallel organisation that would work in full collaboration with the IOC for the good of the Olympic ideal and sport.

These discussions focused on several subjects on the agenda such as “The independence of NOCs”, “Regional Games”, and “The joint development of NOCs”. The NOC General Assembly validated the creation of a Coordination and Studies Commission, consisting of eleven people presided over by Giulio Onesti, and whose members represented all continents. The composition of the commission was thought out in a way that made sure every part of the world was represented, and it was Onesti's wish that commission members be important leaders of the Olympic Movement. Hence, members included IOC figures and NOC presidents such as Konstantin Andrianov (USSR), Gabriel Gemayel (Lebanon), General José Clark (Mexico), Douglas Roby (USA), Tsuneyoshi Takeda (Japan), Hugh Weir (Australia) and Giulio Onesti (Italy), as well as NOC leaders such as Jean Weymann (Switzerland), K. Sandy Duncan (Great Britain), Jean Claude Ganga (Congo) and Henry Corenthin (Mali) (14). The work and draft resolutions put forward and discussed in Rome were all presented to the IOC a few days later, during its session in Madrid in October 1965. In this way, the IOC showed its wish to deal quickly with the question while, simultaneously, informing the NOCs’ GA that it had no decision-making authority with regard to Olympic matters. Onesti and the NOCs underlined the positive stance taken by President Brundage (15)..

However, during the same period, opinion regarding the NOC GA shifted radically. Several members of the IOC's Executive Board and Brundage changed their minds concerning these NOC initiatives. During the preparation of the Tehran GA, and as a result of this evolving balance of power, the IOC President described the Assembly as “a private meeting which does not have the approval of the IOC” (16). With such remarks, Brundage was, in effect, advising NOCs against participating in the event. Furthermore, General Clark, one of Brundage's close associates and member of the Coordination and Studies Commission presided over by Onesti since 1965, who also became member and first Vice President of the IOC Executive Board (EB), differentiated himself from the NOCs. As President of the Association of the Pan American Games, he compelled American NOCs not to attend the NOC Assembly (24). His words resulted in the absence of several NOCs at the GA, as well as during the traditional meeting between the IOC's EB and NOCs’ representatives.

The IOC's Executive Board met in Tehran in May 1967, prior to the official session taking place in the same city. President Brundage pointed out that the IOC Session to be held in Tehran was of considerable importance since the situation, as a whole, was tense. “The international sports federations have just gathered in Lausanne, the national Olympic committees are conducting a meeting in Tehran as we speak, and these two organisations, created by the IOC, criticise the IOC, as do other organisations and the press” (17). Brundage was very clear, and the IOC had no choice but to react. During the meeting, General Clark informed the Board of a letter from Onesti telling him that he had been replaced in the Coordination and Studies Commission by Dr. Josué Saenz, new President of the Mexican Olympic Committee. Had Onesti already sensed that Clark was switching positions? Despite the stances taken by the IOC, the NOC GA in Tehran still gathered 64 NOCs.^8^ The GA and Onesti decided to refer the formal decision on the form of organisation their union should take to the following NOC GA to be held in Mexico City in 1968. The decision was also postponed until the Mexico City GA, given that the IOC President had confirmed he would set up collaboration between the IOC and NOCs. The commission chaired by Onesti was, meanwhile, extended for a further year.

Several members of the Executive Board maintained that the commission chaired by Onesti had exceeded its mandate due to the impossibility of creating an association of NOCs.

The IOC did indeed not leave the question unanswered after 1965, since it set up a mixed commission following the IOC Tehran Session in May of 1967. “The plan was to set up a coordinating commission - called mixed commission—consisting of esteemed IOC and NOC members elected by continent. In addition to annual plenary sessions between the IOC's Executive Board and the NOCs, this arrangement led to the belief, fully accepted by the IOC Commission, that there was no longer any reason for an Association of NOCs to exist” (18).

This period thus became somewhat unclear, according to Lord Killanin, as there was no communication between Onesti and the IOC. However, the work of the NOC GA and its commission was constructive.^9^ According to Killanin, the IOC appeared to have “lost control, there was a conflict of authority, and the two groups were trying to do the same work” (19). Constantin Andrianov, acting in two capacities as Vice President of the IOC and Commission member, referred back to the official mandate given to the Commission. Before the Tehran GA of NOCs, the mission of the Coordination Commission had been to put forward proposals for the future organisation of NOCs. During the GA, the Commission presented two projects for a better organisation of NOCs. The first one expressed the wish to create an association of NOCs (a project backed and driven by Onesti and the Italian NOC). The second proposal was put forward by the British, who suggested creating a standing conference or general assembly of NOCs, in other words, a much more flexible union. In addition, Gabriel Gemayel pointed out emphasised the fact that Onesti “was not acting in a subversive way towards the IOC”.^10^

The NOCs gathered in Mexico City on 2 October 1968, on the occasion of the Games of the XIX^th^ Olympiad. A new wave of international cooperation took place and allowed the statutes of the c to be adopted. The stated objectives of the NOC PGA were primarily to serve the “Olympic Movement in line with the philosophical, spiritual and sporting principles defined by the International Olympic Committee” (20). Promoting and strengthening collaboration between all NOCs was also one of the pillars of this PGA. The three organs of the PGA were the plenary sessions (equivalent to the IOC Sessions), Coordination Commission (that can be considered the counterpart of the IOC's Executive Board), and its President.

The process for appointing GPA members differed from the co-optation system in effect at the IOC since the NOCs themselves chose their representatives. Another difference compared to the IOC lies in the fact that the NOC GPA had its headquarters within the NOC of its president. Consequently, in 1968, the headquarters were in Rome, in the offices of the Italian NOC. Again, the GPA's operating mode, governed by its statutes, shows less centrality than that of the IOC. Emanating from the NOCs, the GPA made democratic representation possible for them, while the IOC offered more of a political representation, ensured by the selection of IOC members.

Onesti was well aware that his name came up regularly during the IOC's Executive Board meetings between 1966 and 1968. The NOC PGA was a matter of debate at almost every Executive Board meeting during these years, and the criticisms and fears regarding Onesti's initiatives continued to grow throughout the period. However, Onesti kept reiterating that the NOCs were the IOC's core supporters and that it is “ridiculous and absurd to claim that we are rising against the IOC, we have no intention of undermining its authority or its power” (Onesti 1968). In his opinion, the opposition was due to purely personal reasons since Onesti seemed to want to put forward reforms for the IOC that were intended to make it, according to him, the world's most democratic and representative institution. Onesti highlighted the IOC's creation, in Tehran, of its mixed commission in charge of contact between the IOC and NOCs. In the Italian leader's view, “this solution is nothing but an improvisation and cannot do much good”. According to Onesti, this commission, which met for the first time in Lausanne, had shown that it could not speak on behalf of all NOCs, since its members, and particularly the NOC representatives, comprising it were not representative enough to speak in the name of their regions’ NOC. The commission created in Tehran was led by Danish IOC member Ivar Vind, accompanied by Dr. Ryotaro Azuma (Japan), Lewis Luxton (Australia), General José de Clarck (Mexico), Syed Wajid Ali (Pakistan), Sir Ade Ademola (Nigeria), Frantisek Kroutil (Czechoslovakia) and Giulio Onesti (Italy).

For all that, the situation remained unclear, and Lord Killanin indicated that several groups had emerged around the NOC PGA with one group aiming to “take over from the IOC, a second one disapproving of the current situation but wanting to know what is happening, and a third one being completely opposed to it” (21). President Brundage let the IOC's Executive Board know of his intention to recommend that all IOC members associated with the PGA hand in their resignation (22). Sir Ade Ademola, on the other hand, underlined the fact that a great majority of African NOCs were in favour of the PGA since it could assist them in their development. Several members, namely Andrianov, held the IOC responsible for the creation of the NOCs’ PGA, with the initiative being the result of meetings between the IOC and NOCs. Andrianov proposed that the PGA become a subsidiary organisation of the IOC to help NOCs, but that the PGA be placed under the IOC's authority, even though he did not see it as a real threat (23). However, for President Brundage “an external organisation is thus of no use and can only create disorder and confusion” (24). Herman van Karnebeek was of the same opinion and went so far as to tell the Executive Board that it should remind Onesti that he was an IOC member for Italy and that he should work for the IOC and not the PGA.

Brundage's discourse and stance regarding the possible union of NOCs rapidly changed between his speech in Rome in 1965 and the end of his presidency in 1972. During these years, the IOC President exchanged a large amount of correspondence with several IOC members on the subject of Onesti's initiative. Studying this correspondence enables us to understand the true position of Brundage and his allies.

### 2.2 Avery Brundage and Giulio Onesti: tension-revealing correspondence

From 1965, much correspondence was exchanged, particularly between Brundage and Onesti, concerning the NOC PGA. The correspondence highlighted numerous issues that did not appear in the consensual records of these organisations’ minutes and official documents. The creation of the General Assembly of National Olympic Committees by Giulio Onesti “angered Brundage, and the group was not recognised for some time” (25).

The Italian first addressed a letter to the IOC President on 1 April 1967, in response to a letter sent by Brundage on 23 March, expressing the fear of some NOCs that an NOC association would concentrate the entirety of the dialogue with the IOC. Onesti contested this point of view and reminded him, “once again, that the association does not intend to replace, nor could it, the direct representation power of every NOC with the IOC” (26). By writing these lines, Onesti was only reminding Brundage of the need to respect each NOC's Olympic sovereignty. For that purpose, the association's statutes intended to limit its role to mere recommendations and proposals for the IOC. According to Onesti, many people refused to understand the significance and goals of the proposed association. In a letter, Brundage had also informed the IOC's Secretary General, Johan Westerhoff, that Onesti's initiative was purely personal, a statement rebutted by the Italian in a letter sent to the Secretary General urging him, as he always did, to attend the NOC GA and observe its works (27). Onesti also sent a letter to all IOC members to clarify the situation, arguing that the NOCs had never intended to replace the IOC but that.

The Mixed Commission, chaired by Vind since its creation in 1967, was dissolved at the 68th IOC Session in Mexico in 1968. It was replaced by a Coordination and Management Commission for IOC/NOC Relations, presided over by General José Clark and composed of five mixed commissions. It is important to remember that among Brundage's friends within the Olympic Movement was the Mexican General Clark, “a man of Brundage's calibre in many respects” (28). With Clark heading this commission, Brundage had his best ally for foiling Onesti's plans. For Onesti, however, the most striking accusations were those regarding the funding of the PGA's activity. The PGA headquarters were at the Italian NOC in Rome, and Brundage implied that the funds made available to the PGA came from the “Totocalcio” and the Italian NOC.^11^ Onesti informed Brundage that the PGA's funding came from donations made by the NOCs themselves and private individuals or companies (29). These letters between the Italian and the American stirred up controversy, and tensions threatened the unity of the Olympic Movement. The difficulties encountered during that period had to be overcome for the good of Olympism.

Alongside his exchanges with the IOC however, Onesti received the support of other European personalities involved in the Olympic Movement. Marceau Crespin, for example, contacted Onesti to share his remarks regarding the plan to create an association of NOCs, which could have positive results. From a general point of view, this future association, where all NOCs would have equal representation, should allow those insufficiently represented within the IOC (particularly the African and Asian NOCs) to make their voices heard.^12^ This could “make the mostly Anglo-Saxon narrow and conservative structures set up by the IOC progress” (30). Still showing aversion to the Italian's initiatives, Brundage sent a circular to the NOCs and IOC members that sounded like a call to order and a clarification (31). In this letter, he talked about the misunderstanding of several NOCs, using the Nordic NOCs as an example, since they had questioned the IOC regarding its headquarters. The five Nordic NOCs went so far as to prepare a memorandum asking if the IOC's headquarters were in Rome, Paris, Chicago, or Lausanne. Were all the initiatives implemented during the 1960s and 1970s, particularly those led by the NOCs, not modifying the world map of Olympism, and more particularly the original dissemination centres for Olympism? Indeed, Rome was home to the NOC GPA and Paris to the GA of European NOCs, Brundage's offices and place of residence were in Chicago, and the IOC's headquarters were in Lausanne. Brundage firmly responded to the question by saying that “there is only one answer to that question, and it is formal. The IOC's headquarters are at the Château de Vidy in Lausanne. The PGA of NOCs, set up only recently, is not recognised by the IOC and ‘will never be recognised as the mouthpiece of the NOCs since a majority of them would rather address the IOC directly, not through an intermediary’ (32). This letter mentioned an article published in the magazine *Jeune Afrique* (Young Africa), discussing the wish of the Soviet leaders for a complete overhaul of the IOC, particularly regarding the direct representation of the NOCs and IFs. Was the Cold War gatecrashing the Olympic Movement led by the IOC and presided over by the American Avery Brundage? (33) This proposal would amount to creating “a real sports United Nations that would paint a more exact picture of the sporting forces involved. The African NOCs supported such a project. With only four votes within the IOC at present, Africa would be entitled to have 35 representatives. It would thus carry weight in the decisions of a new IOC. Justice would be done” (34).

This article was used by Brundage to provide evidence of the reform initiative of Onesti and his PGA of NOCs. Brundage made use of the article by integrating it into his letter to discredit Onesti in the eyes of the NOCs. The PGA of NOCs and GA of ENOCs were also in favour of a better representation of NOCs, hence the setting-up of a general assembly where each NOC had a vote.

Brundage's consistent opposition seemed to “be fuelled by his conviction that Onesti wanted to replace him as IOC President even if, as noted by Italian newspaper *Il Messaggero*, ‘Onesti had neither Brundage's money, nor his pride or interest in the presidency’ (35). Moreover, Brundage seemed to have removed those of the IOC's important figures who saw Onesti and his initiative as a good way to serve the Olympic ideal. This was also shown by certain opinions on the matter of Dutch member Johann Westerhoff, who was IOC Secretary General from 1967 to 1969 and who was linked to the first NOC meetings. His relations with Brundage over the two years were indeed tense, and although Westerhoff did “important restructuring work vis-à-vis the IOC, managing the acquisition of the IOC's new offices in Lausanne (..), he was deemed too direct and progressive for the IOC” (36).

A letter addressed by Westerhoff to a Swiss newspaper provided information regarding Brundage's aversion towards the NOCs’ initiatives and the tactics he used to halt the action of those actors, primarily Onesti, who were in favour of NOC emancipation: “Later on, I was categorically banned from speaking to the representative of the NOCs (Giulio Onesti) and the federations (Roger Coulon). As official communication was forbidden, I did not insist. The dislike between the two associations was huge. I thought that creating an organisation of NOCs could only be beneficial to the IOC and Olympic ideal. The same for the federations (..) Everything was done to torpedo the NOC session before the 1968 Games in Mexico City. Or at least to make it impossible to find a conference room. On the subject, Brundage said: IOC's Vice President José de Clark already knows what he has to do to stop them from meeting”.^13^

Throughout the 1960s, relations between the IOC and the NOCs were tense, particularly towards Onesti and his initiative to create an association of NOCs. Three years later, the French Olympic Committee and its President Count Jean de Beaumont took the initiative of inviting all European NOCs to Versailles with the aim of laying the foundations of a future association of European NOCs.

## 3 The difficult institutionalisation of the AENOC (1968–1975)

### 3.1 The versailles turning point in 1968

Under the leadership of Onesti, the union of NOCs was being echoed by its French neighbours. When the Italian found out that it had been decided, during a meeting of the French NOC, to create an association of European NOCs, he contacted his friend and President of the French Olympic Committee, Jean de Beaumont.^14^ According to Onesti, it was somewhat “ridiculous that Europe should be so far behind the other continents since, among others, the initiative of some European NOCs regarding the organisation of European Regional Games could not be efficient without the creation of such a body” (37).

According to him, European interests were not sufficiently represented within the NOC GA. The NOCs of other continents had indeed already united to “make their voices heard by both the IFs and IOC, especially through the African Sports Council with Jean Claude Ganga and the Pan-American Federation with José De Clark, yet nothing comparable existed in Europe” (38). It should be noted that the other continental institutions were primarily designed to organise regional Games and to promote Olympism on other continents.

Danet reiterated that all the Olympic figures involved in the NOC GA, who were mostly European, had shown that “the main purpose of their mission was to collaborate with the IOC, in the interest of preserving the OM, despite the exaggerated aspirations of IFs and some overly politicised NOCs” (39). The aim of this French initiative was therefore, through the creation of an association of European NOCs, to promote a voice and European reality that would manifest themselves during these sessions where only European NOCs would be present.

Thus, upon the invitation of the French Olympic Committee, 22 European NOCs^15^ were present or represented for a first contact at the Trianon Palace in Versailles on 7 and 8 September 1968. Among the ENOC representatives, 8 were IOC members^16^ in 1968. The presence of 13 International Olympic Federations^17^ should also be noted. Following the participants’ unanimous request, Count de Beaumont, President of the French NOC and IOC member, assisted by Alain Danet, Secretary General of the French NOC, presided over the meetings. The agenda of the first session of European NOCs featured 5 major topics: creation of a cooperation consortium between European NOCs; better coordination between the IOC and NOCs; participation of European NOCs at the third NOC GA in Mexico City; European Games project; and the Olympic Congresses.

During the meeting in Versailles, the ENOCs became aware of a project, jointly developed over several months by the public authorities of the cities of Mulhouse (France), Freiburg (FRG) and Basel (Switzerland). The aim of this bid, based in the Rhineland region, was to organise the first European Games. Committed to respecting the authority of the IOC, the European NOCs offered to “contribute, by means of amicable cooperation, to the growth of the Olympic Movement based on friendship, fraternity and love for universal peace and an efficient guarantor of a better future for the world's youth” (40). During this meeting, Gafner also suggested that a recommendation for the organisation of a future Olympic Congress be submitted to the IOC in the following years with the aim of reconnecting with this Olympic tradition.^18^

But ultimately, was the aim of this NOC Session in Versailles not to lay fertile and useful foundations for the ambitions of Count Jean de Beaumont and his allies? It can in fact be considered that one of the main objectives of this first European NOC meeting was also to lay the groundwork for Count Jean de Beaumont's candidacy for the IOC presidency in 1968 but, above all, for that of 1972. Some archives hinted at the fact that, beyond having encouraged the first meeting between ENOCs, “it should not be forgotten that Count de Beaumont, as a generous host, sought to have his own ambitions within the IOC approved by campaigning against Brundage; that Onesti encouraged the ENOCs to make a preliminary decision about establishing an association of worldwide NOCs; that Gafner acted as a spokesperson for the creation of a specific association for ENOCs and European Games; that Mollet tried to lay the foundations for this in 1974 and enhance his own image by displaying the principles of a new style regarding the IOC's administration; or that the representatives from the socialist ENOCs, whose strings were pulled by Wieczorek, could calmly observe the process as long as they continued to receive the help of the egocentric struggle for power of Beaumont, Gafner, Onesti and Mollet. Some opposition towards the IOC's Secretary General was clear” (41). This excerpt from the archives of the DOSBwas taken from a report of the Versailles meeting, written by the representatives of the FRG NOC who attended. The report brought to light the personal stakes behind this first symbolic and strategic meeting.^19^ The promoters of the meeting found themselves at the crossroads of these stakes. Beaumont wished to use the meeting to promote his project. Onesti was the founder and president of the NOC PGA, and the support of the ENOCs was crucial in establishing an association of NOCs. The European Games project was important for the creation of a continental association and Gafner had elected himself its spokesperson. Mollet, on the other hand, was a central figure in the history of the Olympic Movement. The games they were playing could be seen in the debate transcripts of the DOSB.

President Beaumont submitted a project regarding the creation of an ENOC task force intended to enable a first active collaboration and prepare work with the IOC's Mixed Commission (42). The operational nature of this second meeting led the actors to reveal their intentions, in favour, against or cautiously neutral, even on standby, vis-à-vis the project. Opposition rapidly emerged with the intervention of Finish leader Erik Von Frenckell, who also represented the Norwegian, Danish and Icelandic NOCs at the meeting. He pointed out that neither his NOC nor the ones he represented would be willing to join this new association and would rather act as observers (43).

Mollet, the Belgian, found it unfortunate that the Scandinavian countries remain mere observers, but insisted on the need to create a task force as quickly as possible and suggested that France take its presidency through Count de Beaumont (44). Mollet also insisted on the fact that the members of this group should not be part of the IOC's Mixed Commission. This modality guarded the new group against any form of infiltration. During this meeting, the representative for the Spanish NOC, Juan Antonio Samaranch, endorsed Beaumont's proposal and asked that a permanent liaison committee between European NOCs be established. The members present in Mexico City in 1968 thus decided to create a task force between European National Olympic Committees under the presidency of Count de Beaumont.

The first task force was thus created in Mexico under Count de Beaumont's leadership and included Jean Waymann (Switzerland), Raimundo Saporta (Spain), Epaminondas Petrialas (Greece), Igor Kazanski (USSR), Nebojsa Popovic (Yugoslavia), and an observer, Sten Svensson (Sweden). Four substitutes were also appointed: Helmuth Behrent (GDR), Iolanda Balas (Romania), Claude Collard (France), and Emmanuel Bosak (Czechoslovakia) (45). The president invited Mrs Nadia Lekarska (Bulgaria) to participate in the working group on women's sport.

The GA of ENOCs, held in Dubrovnik in 1969, saw the election of Beaumont and Weymann as President and Secretary General respectively. The latter were determined to forge ahead and end a temporary situation that had lasted too long by establishing a permanent institution. To do so, Beaumont and Weymann addressed a letter to every European NOC to introduce the future association of European NOCs. A non-exhaustive list was presented, recapping the missions and aims of the association: protection and development of the Olympic ideal and movement; better cooperation, collaboration and understanding between ENOCs; defence of the NOCs’ own interests; development of European solidarity; study of the possibility of organising European Games or Youth Games; pooling NOC efforts, etc (46). One of the reactions to this circular commanded attention since it came from the spokesperson for the NOCs. Indeed, Onesti responded to the letter by sending another circular as President of the Italian NOC and NOC PGA to the presidents and secretary generals of all European NOCs. In this letter, Onesti presented his opinion on the creation of a group of European NOCs and on the project of European Games. The Italian welcomed the initiative of a closer relationship and friendly collaboration between European NOCs but did, nonetheless, express some reservations.

According to him, it was not appropriate to create an association of European NOCs with the consequences it would entail (affiliation, statutes, election of organs, bureaucratic apparatus, etc.) (47). Europe's position was unique, and it seemed unnecessary to create an institution of ENOCs merely to repeat what was being done on the other continents. Continental institutions such as ODEPA (Pan American Sports Organisation), ODECABE (Central American and Caribbean Sports Organization) or the Asian Games Federation, were solely or largely based on the recurring organisation of continental games. Europe's case was completely different, as Onesti had already pointed out during the sessions in Mexico City Session and Dubrovnik.^20^ According to him, the circumstances at the time were not conducive to creating continental games and, especially considering the reservations expressed by various NOCs, it seemed advisable to suspend the project of creating European Games (48). However, for Beaumont, Gafner and all the other advocates of the European Games, the organisation of such an event would cement Olympic solidarity between European NOCs and unite their actions. For Onesti, on the other hand, the project represented the main obstacle for concrete European Olympic cooperation. Opposition to the project came particularly from IFs, mainly the athletics and swimming ones.

Lastly, Onesti considered that organising European Games would primarily result in a conflict with the IFs. If the ENOCs were to antagonise the IFs, the relationship between the NOC PGA and the IFs was likely to be tarnished for a long time. Onesti recommended strengthening NOC-IF links vis-à-vis the IOC rather than the creation of an additional intermediary with an association of European NOCs (49). Was Onesti not seeking, first and foremost, to promote his personal project of creating an association of all NOCs worldwide before one of European NOCs?

The first signs of dissent began to appear vis-à-vis the AENOC, as shown by a letter from Swiss Gafner to Onesti, Mollet and Wiezorek. This letter was a response to the circular sent by Onesti to the European NOCs. In this letter, Gafner mentioned several differences of opinion regarding Onesti's positions. Disagreements emerged between the Swiss and Italian leaders concerning the status of Olympic athletes and Olympic Solidarity. The most notable disagreement drawing attention was related to the possible union of European NOCs. The convictions of these important figures, hitherto closely linked, differed for the very first time, to such an extent that Gafner deemed it “essential that we have a discussion among ourselves before we face the GAISF (Global Association of International Sports Federations)” (50). The Swiss could not understand why President Onesti appeared to “fear a meeting of European NOCs. Is he afraid of competition for the NOC PGA or is it a matter of prestige between two important European NOCs which, for me who represents a smaller NOC, would not be acceptable?” (51) He was undoubtedly referring to the fact that the French Olympic Committee and its President Beaumont had initiated this union of ENOCs. Gafner warned against a potential rivalry between the NOC PGA and the European NOCs. Indeed, if the links between both institutions boiled down solely to putting “obstacles in each other's way, then I am not willing to participate in this little game” (52). The Swiss also sent a personal letter directly to Onesti to share his thoughts with him. Onesti saw a difference between the future association of ENOCs and the already existing associations on the continent (53). Gafner failed to understand the need to differentiate between continental NOC associations, since the latter could slowly become branches of the NOC PGA (54). The President of the Swiss NOC did however agree on one point, that the project of European Games faced many obstacles that were truly hard to overcome. One particular element appeared to displease Gafner who admitted “seeing Onesti's recent negative position as a hostile gesture. President Keller (GAISF) has mentioned this in the letter recently addressed to the NOC PGA. Once again, we seem to enjoy displaying our differences to the IFs, who got more than they had hoped for. Naturally, differences of opinions can occur between us. It does, however, seem clumsy to expose them to those who remain, for now at least, partly our opponents” (55). The NOCs, and particularly European NOCs, should show their unity rather than their differences, particularly in front of the GAISF.

This exchange brought to light the theory that Onesti's position towards the AENOC was an ambiguous one. Yet, the initiative to unite European NOCs was fully consistent with the dynamic he had set in motion back in 1965. In this regard, when researching minutes from the NOC GA and NOC PGA, only a few references could be found to the ongoing association of European NOCs launched in 1968. Coincidence or intentional? Comparing official reports with correspondence makes it possible to perceive Onesti's ambivalent position.

In the midst of this exchange of correspondence, Onesti would take advantage of the 1970 ENOC in Munich to refer back to the circular addressed to the ENOCs. Gafner's remarks seemed to have been heard by Onesti since the Italian began his speech by saying that it was now necessary to have some form of cooperation between ENOCs, and that “it is useful to adopt a flexible procedure. But it is for the European NOCs to decide, and I will give my full support to the idea that will be expressed during this meeting” (56). However, uniting the points of view regarding the future union of European NOCs still proved difficult.

### 3.2 European Olympic unity remains laborious

Following the first meetings between the ENOCs in Versailles, Mexico City and Dubrovnik, Count Jean de Beaumont submitted the blueprint of the statutes for the “European Union of National Olympic Committees—EUNOC” to Luc Silance, Secretary General of the Belgian NOC, in a letter asking for his feedback (57). This preliminary draft of the EUNOC statutes was to be sent to all European NOCs as a working basis for the beginnings of European Olympic cooperation. According to the blueprint, the goal was to develop and promote friendly relations between the different European NOCs. The EUNOC intended to serve the international Olympic Movement within the frame set by the IOC (58). The association was open to all European NOCs recognised by the IOC and should strive, as a priority, to foster relations between ENOCs, promote research regarding physical and sporting education and encourage the development of sport in full respect of the Olympic Charter. The EUNOC included three bodies: the plenary session, the executive board, and the president. Each member NOC was entitled to one vote. The EUNOC headquarters were housed within the offices of the current president's NOC, who assumed the association's running costs. The EUNOC official languages were French and English.

The annual ENOC GA took place in Munich in 1970 and Secretary Weymann read out a letter sent by the GDR NOC Vice President, Günther Heinze.^21^ Heinze wished to remind the members present that the creation of a union or association of ENOCs was “not only for the purpose of organising European Games, but also for other important tasks “ (59). This clarification regarding the scope of application of an ENOC union, conveyed in Heinze's letter, was strategically important since, for many ENOCs, like the British and Scandinavian, the future union was based solely on the organisation of European Games. This point was the main issue delaying the institutionalisation of European NOCs. Belgian leader Luc Silance also wished to reiterate the goals and missions of this future ENOC association. In line with the statements of Belgian President Raoul Mollet, Silance insisted on the need for a group, an association, or a union of ENOCs. The aim was to define a common European Olympic point of view. As he stated, “we have never had a European group.^22^

Solidarity between European NOCs should be put into place, yet in order to organise and materialise this solidarity, there needed to be a place and, until then, that place was the annual session of ENOCs. In the same way as Heinze, Silence underlined the fact that European Games would be only one aspect of this union, and that the number one facet was and had to be a demonstration of European Olympic solidarity.

After all, what would the group's objective be if not to encourage relations between European NOCs? The Norwegian NOC reiterated that the Olympic Movement was a worldwide movement and that it would be a mistake to divide it into continental associations (60). The main aim was to know if European NOCs should meet to gain better representation within the IOC. A union of European NOCs would serve to reinstate Europe at the centre of the Movement. This point was often subject to debate. The Secretary General of the Czechoslovakian NOC, František Kroutil, endorsed the idea that the ENOCs had a particular role to play regarding the challenges that Olympism had to meet in the years to come but did not think that a rigid structure, defined by statutes, was essential. By way of example, Dr. Van des Ploeg, Secretary General of the Dutch NOC, wished for the continent to remain focused on European problems instead of relying on historical considerations. If Europe leaned solely on its history and role within the Olympic Movement, it could be criticised for having a vision that was too conservative (61).

The GA gathering ENOCs in Munich brought to light a certain ENOC geography, with those in favour of an association on the one hand and the advocates of a more flexible union on the other. A group emerged around the Yugoslav, Scandinavian, Soviet, British, Austrian, Czechoslovakian and Hungarian NOCs as they were all favourable to the establishment of flexible cooperation in the form of conferences examining the issues regarding the Olympic Movement in general. Conversely, the French, Belgian, Swiss, West German, and Italian NOCs were in favour of a more formal union.

It was therefore necessary for the continent to reach a compromise between the Belgian proposal and that of its allies, and those of the aforementioned NOCs. Facing the need to reach this compromise, Swiss Olympic mediator Gafner stressed the importance of finding common goals that would unite the ENOCs. It seemed “best to take little steps all together rather than a big step that divides us” (Gafner 1970). In this perspective, discussions should not be limited to purely European topics. The Olympic Movement was international and consequently there were no specifically European problems.

Initially presented as an instrument for the emergence of European solidarity through sport and as the flagship project to cement the union of ENOCs, the European Games was a topic that divided the ENOCs instead of uniting them. As one of the project's spokespeople, Gafner felt that the project had not yet matured and thus remained unfeasible in the short term. A lead that was investigated was the possibility of creating an Olympic event focused on the young and European Olympic hopefuls. In the end, two sensitive issues caused the institutionalisation of an ENOC assembly to drag on. On the one hand, the more or less structured, more or less autonomous organisation the ENOC group should take and, on the other, the controversial organisation of European Games.

### 3.3 Opposition from the British NOC

Another ENOC GA also led to several confidential letters being exchanged, that of Monte-Carlo in 1973. For the first time, Lord Killanin attended as the IOC's new president, and correspondence was exchanged prior to the GA between Killanin and K. Sandy Duncan.^23^ The British Secretary General challenged the IOC President regarding the European Olympic organisation. Duncan was opposed to the creation of an association of European NOCs. Indeed, since the first session of European NOCs in 1968, the British had always shown reluctance concerning the creation of an association of European NOCs. Duncan mentioned a host of threats for the IOC if such a European institution was to see the light of day, “Without the IOC's help, what the true role of this body could be: this is what we should examine at Monte Carlo. From what I understand, the NOC PGA is likely to disappear and be replaced by a similar organisation led by the IOC from Lausanne. What is the IOC's policy on these continental NOC groups, which could all too easily become “lobby groups”? (…) Is there not the risk that instead of a bulky organisation like the NOC PGA, there will be four or five “continental lobby groups” (62). Were these remarks not a concealed way of scaring and warning the IOC? According to Duncan, the NOCs’ continental organisations were “lobby groups” applying pressure on the IOC. He put forward the same concerns and talking points as the IOC's former president, Brundage. Nevertheless, in his letter, Duncan mentioned a central concern for the future AENOC, that of the IOC's recognition, for such an organisation to be able to conduct its Olympic business in Europe.

Duncan's letter was rather critical regarding the future association of European NOCs. Feeling that manoeuvres were being made behind the scenes, and at the request of the ENOC GA's President, Jean Weymann addressed a letter to the IOC President, Killanin to present the areas of reflection that would be discussed during this important GA in Monte Carlo (63). First, the European NOCs would have to define the activities of the ENOC GA. A question was consequently asked, “is it advisable to give ENOCs an important role within the organisation of the Olympic Movement or should it remain merely an organisation for reflection and exchanging ideas?” (64). This letter reminded the IOC President that the African, Asian and American NOCs had already set up continental organisations. The latter were founded on the organisation of continental games. The European case was, however, much more complex since two trends had emerged within the European NOCs. On one hand, some ENOCs advocated an ENOC GA that would be “merely an unofficial and friendly organisation”, while on the other, as in the case of the GA presidency at the time, some defended the idea of a more official organisation, capable of drawing up a common European Olympic policy. This also represented the opportunity for Beaumont and Weymann to put back on the table a subject that was dear to many founding European NOCs, that of the European Games. Within the ENOCs, members were conscious of IF opposition to the project, yet several ENOCs wished to consider the solution of European games dedicated to under 23s. The aim was also to discover new European talent. If this project was accepted, the GA would have to decide on a host venue. Weymann underlined in his letter that it was not necessary for such games to take place in one single city but that “the symbolic application from Basel, Freiburg and Mulhouse should be reconsidered” (65). As a result, reactivating the project not only required IOC backing but also validation from the Olympic IFs. The composition of this future European Olympic organisation also needed to be considered. Mollet from Belgium had always wished that the organisation's members would not be members of the IOC or IFs. At Monte Carlo, it was necessary to discuss whether or not the President of the ENOC GA and its members could also be IOC members.

This British opposition is not unlike the British position on European integration. Indeed, the opposition between the ‘federalists’ and the ‘unionists’ seems to be reflected in the construction of the European Olympics.

### 3.4 The turning points of the Monte Carlo and Paris GAs

The ENOC GA held in Monte Carlo in 1973 marked a further advance for European NOCs. It was necessary to consolidate this union “by creating a bureau representing European trends, (…) Our overall position will have, in Varna or elsewhere, more weight than our 32 voices expressed individually” (66). Elected in 1972 as the IOC's new president, Lord Killanin attended the GA.

Prominent figure Raoul Mollet presented a report on the actions and conceived role of the ENOCs during the GA. He drafted an ENOC profile, and recognised what they represented within the modern sporting world, as well as their place in Olympism. He thus put forward two proposals to the GA. Firstly, that a firm stance be taken for or against a formal and structured cooperation between European National Olympic Committees. Secondly, that the best way to provide ENOCs with a rational organisation be studied. The ENOCs in attendance approved Mollet's report and proposals. They therefore needed an organisation, a forum for European discussions, particularly regarding Europe's specific issues. Following Mollet's report on the possible creation of an association of European NOCs, the assembly agreed to set up a body consisting of eight topic-based working groups and a president, Raoul Mollet. The representativeness of participants in the ENOC working group titled “Projects and Studies” symbolised the collective awareness of the group's leaders during the Monaco session in May 1973. The group's main purpose was to study, draft and submit, to the V^th^ GA in 1974, a viable option for developing better cooperation between all European National Olympic Committees. Between the 1973 and 1974 GAs, this group, chaired by Mollet, would meet four times (in Vittel, Varna, Brussels, and Vaduz).

On 23 June 1974, 80 years to the day after Coubertin's speech marking the official creation of the International Olympic Committee and the revival of the modern Olympic Games, the ENOCs gathered in Paris for the V^th^ annual assembly. This particular ENOC GA represented a true landmark. The working group “Projects and Studies” seemed to favour an organisation capable of tackling specific issues “without encroaching on the authority of the IOC and without troubling the NOCs of the other continents” (67). Beaumont, as president of this “permanent ENOC consultative conference” was the promoter of this GA in Paris. He considered handing in his resignation, as he felt he had accomplished his mission but “wants the presidency to remain French” (68). Danet presented this group as being an unclearly defined committee until 1974. With small steps, and after a long preparation period, an Olympic Europe was formed thanks to the action of the ENOCs and several of its members, as well as to sport (69). The newly founded French National Olympic and Sports Committee (FNOSC) welcomed this new GA, and its president, Claude Collard, declared himself in favour of setting up an organisation of NOCs in Europe, flexible enough for the NOCs to feel free but structured enough to be effective. Its representativeness was important since the organisation should reflect all the ways of thinking that were driving Europe, and should have an inclusive and universal structure encompassing all ENOCs, so that all Europeans may recognise themselves in this organisation (70).

Mollet proposed that the executive board be composed of seven people: a president, vice president, secretary general and four members. The secretary general of the Swedish NOC, Bo Bengston, publicly displayed a shift in position when he pointed out that “we were rather hesitant a while ago on the need for highly organised cooperation among ENOCs. In fact, we were in favour of *ad hoc* consultation or meetings, when they proved essential. After the Varna Congress, however, we became convinced that a stronger structure, as well as sustained and continuous cooperation among the European Committees are necessary” (71). Several ENOCs with similar views to that of the Swedish NOC in favour of annual consultation only realised, at the Varna Congress, that it had become vital for the ENOCs to meet within an organisation. For Alain Danet, while, prior to Varna, a light structure could be accepted, this was no longer possible after the 1973 Olympic Congress. The Varna Congress was poorly prepared by the NOCs and a complete failure. Compared to the IOC and FIs who were much better prepared, the NOCs lacked organisation (72). The FIs had coordinated their opinions at the meeting of the GAISF in Oklahoma. Professor Vladimir Gernusak, for example, vice president of Czechoslovakia's NOC, affirmed that certain federation representatives went so far as to place the IFs on the same level as the IOC, while considering the NOCs as mere “second-rate organisations with limited importance” (73).. The IOC, nonetheless, showed signs of moving towards the NCOs when it refused bipartisanism with the IFs and, following the Varna Congress, maintained the tripartite commission with numerous new opportunities, including working with governments, athletes, mixed commissions, and debating the place of women.^24^ This should be seen as a form of recognition towards the NOCs.

The British also realised that “the NOCs no longer really knew where their place was in Varna”. It was imperative that both the NOCs and ENOCs unite their voices to rebalance the IOC-IF-NOC triptych. Collard and Mollet were in favour of such a structure and immediate action. Mollet consequently put a fundamental question and the proposal to have a bureau composed of a president, vice president, secretary general and 4 members was accepted.

It was the proposal put forward and backed by Mollet that gave rise to the most debate concerning the composition of the bureau. The working group suggested that no IOC member, or IF president, vice president or secretary general be electable to the ENOC Bureau. Through such a proposal, were the working group and Mollet seeking independence in the future association's governance while avoiding a conflict of interest with the IOC? Or was it a way of distancing certain European members of the IOC from the organisation? According to a hypothesis that emerged, it was perhaps a way of removing Jean de Beaumont from the presidency and allowing Mollet to replace him, given that the Belgian was not a member of the IOC. Of the 27 voters, 12 were in favour of the proposal, 13 against, and 2 abstained. The proposal was therefore rejected.

Immediately afterwards, the first executive bureau of the ENOCs was elected. There were three candidates for the presidency: Count Jean de Beaumont (France), Bo Bengtson (Sweden) and Dimitriy Prokhorov (USSR). The Soviet, however, expressed his support for the French candidacy, “we feel that the history of Olympism owes much to France. On the 80th anniversary of Olympism, it would in fact be highly unfair to envisage any other candidacy than that of a representative of France. We consider that it would be an error not to support the candidacy of Count de Beaumont as President of the Bureau” (74). He thus decided to withdraw his candidature in favour of Beaumont, and Beaumont was elected in front of Bengtson.^25^ Prokhorov was elected Vice President, and Swiss candidate Jean Weymann Secretary General. As soon as Beaumont became president, no other French person could be member of the Bureau, and Bo Bengtson (Sweden), Lia Manoliu (Romania), Janusz Piewcevicz (Poland) and Peter Ritter (Liechtenstein) were elected members of the Bureau (75). Gathered in Paris, the ENOCs thus gave themselves a legal structure which replaced the somewhat informal general assembly (76).

Count de Beaumont had admitted to Killanin that he had no intention of running again for president at the 1974 ENOC AG in Paris. However, when the plan to provide the ENOCs with a structure was adopted by a majority at the General Assembly, he changed his mind. According to Beaumont, it was his duty to run again so as to reduce the risk, in his words, “of any ambitious plans” and “maintain this organisation within the confines of the Olympic Movement and under the control of the International Olympic Committee” (77). He finally understood that without the recognition of the IOC, the organisation had no future.

The work of the groups led by Mollet, which was carried out in relation to the Paris AG in 1974, laid new stones for the ENOC organisation, which led to the approval of the Statutes of the Association of the European National Olympic Committees (AENOC) a year later in 1975.

Each European NOC, recognised by the IOC, could become a member of the AENOC while remaining independent. Three organs made up the association: general assembly, executive committee, and commissions. The ordinary general assembly was held every year and each ENOC member had one vote (78). The seven-member executive committee was elected by the GA for the duration of an Olympiad. The commissions represented the third organ of the AENOC. The executive committee could suggest that the GA set up commissions on particular topics, with the aim of conducting investigations and appointing expert members (79).

## 4 Conclusion

The period between 1960 and 1970 was a pivotal moment in Olympic history. The IOC saw the end of Brundage's twenty-year presidency, and his succession sparked much interest. The IOC faced many challenges, such as the increasing intrusion of politics into the Olympic Games, the growing scale of the Games, the commercialisation of sport and increasing demands from NOCs and IFs. The NOCs want to participate actively in promoting Olympism in their territories and no longer be simply organisations responsible for selecting delegations to participate in the Olympic Games. This period truly marks a renewal of the NOCs in Olympic history.

In this context of multiple rivalries, certain alliances were formed within the NOCs, particularly in Europe, but also within the IOC. Beyond the criticisms of Brundage and his associates, the ENOC disagreed in particular on the form that this future association of European NOCs should take. The main aim was to bring together the European NOCs within an Olympic Europe without isolating the old continent from the Olympic Movement, which remains global. This union aims to create regional and continental Games, promote Olympism and serve certain personal interests. Among the ‘unofficial’ objectives behind the creation of ANOC, this initiative can be presented as a desire to create a ‘European Olympic lobby’ to maintain Europe's central position. However, it was not until 1975 that the Association of National Olympic Committees of Europe was created, replacing the somewhat informal annual assembly of the ENOC. The period from 1965 to 1975 was therefore marked by sometimes tense debates between those in favour of a formal association of NOCs and those in favour of an informal union. This made it difficult for ANOC to move from the debate phase to concrete actions and missions. From 1972 onwards, with the arrival of Lord Killanin as IOC President, relations between the IOC and ANOC/AENOC evolved somewhat towards a cordial understanding, even though the Irishman still did not recognise AENOC.

The unity of the global Olympic Movement was threatened in the 1960s and 1970s, with the NOCs wanting to unite in associations to have more influence over IOC decisions and the International Federations wanting to come together in one institution. President Brundage saw this as an attempt to undermine the authority of the IOC, to bypass it or to put pressure on it to demand a larger share of the TV rights that were developing around the Olympic Games.

In 1980, with Juan Antonio Samaranch becoming president, a new phase of consolidation and improvement began for the Olympic Movement. Samaranch always positioned himself as a defender of the NOCs. He demonstrated this from the very first day of his election as IOC President, recognising the role of the continental associations and expressing his willingness to collaborate with them. Before him, the continental associations were merely tolerated by the IOC and received no assistance. He wanted to involve the NOCs in his policy and did the same with the international federations. Indeed, the IOC satisfied the Olympic IFs with the creation of the Association of Summer Olympic International Federations (ASOIF) in 1983, the Association of International Olympic Winter Sports Federations (AIOWF) in 1976 and the Association of International Sports Federations Recognised by the IOC (ARISF). It was then, at the instigation of IOC President Samaranch, that the IOC granted recognition to ANOC in 1981 and subsequently to AENOC.

It should be noted that all continents had their own NOC associations during those years. However, the history of the European NOC association is unique within the Olympic Movement and differs from other continental NOC associations that were created to organise regional and continental Games.

## Footnotes

^1^This Research is Part of the International Olympic Committee's (IOC) 2023 Research Grant Programme for PhD students.

^2^Secretary General of the Swiss Olympic Committee from 1946 to 1981 and Secretary General of ACNOE from 1969 to 1989.

^3^Honorary Secretary General of the French Olympic Committee from 1962 to 1968.

^4^First President of the French National Olympic and Sports Committee in 1972, until 1982.

^5^President of the Belgian Olympic and Interfederal Committee from 1965 to 1989.

^6^Within these Various Archive Collections, it is Important to Distinguish the Private Circulars from the ‘Public’ Circulars Addressed to Enoc Members, and Conduct a Comparative Study of the Arguments Developed, on the One Hand in the Private Correspondence and, on the Other, in the Minutes of the General Assemblies and Working Groups, in Order to Identify What Differentiates them and Decrypt the Political or Sporting Dynamics or any Underlying Aims.

^7^President of the Italian NOC (INOC) and IOC member.

^8^Of which 23 were European, 18 Asian, 15 African, 7 from the Americas, and the Australian NOC. Compared to the GA in Rome (1965), there were 4 European NOCs and 6 American NOCs missing. The Australian and the 15 African NOCs were present at both GAs, and there were 6 Asian NOCs more than in 1965.

^9^IOC Board member.

^10^Gabriel Gemayel is Member of the Board and Committee of Ten.

^11^The Totocalcio is the Italian sports lottery, at this time, the Italian NOC was entirely financed by Totacalcio and did not receive any public funding from the Italian government.

^12^Colonel Marceau Crespin (1915–1988) was namely executive officer for Olympic preparation in 1961 and director of Youth Affairs and Sports from 1967 to 1974 in France.

^13^Article by Johann Westerhoff, Swiss journal “Sport” dated 13 or 19 March 1969, Westerhoff Collection, F-A01-DS/027, Archives of the IOC's Olympic Studies Centre, Lausanne, Switzerland.

^14^To date, in the archives studied, we have found no prior potential dialogue between Onesti and Beaumont on the calling of an assembly of European NOCs in 1968.

^15^GDR (Heinze, Behrendt), FRG (Dr. Lotz, Gieseler), Austria (Fried), Belgium (Mollet, Prince de Merode), Bulgaria (Stoytchev, Mateev), Spain (Lopez), France (Danet, Dr. Carle, Collard), Great Britain (Duncan), Greece (Patralias), Hungary (Csanadi), Italy (Onesti, Garroni, Martucci), Luxembourg (Link, Hentges), Monaco (Milo), Netherlands (Dr.vd Ploeg), Poland (Reczek, Wieczorek), Portugal (Correa Leal), Romania (Siperco), Sweden (Svensson), Switzerland (Gafner), Czechoslovakia (Kroutil), USSR (Savvin, Kazanski), Yugoslavia (Popovic, Takac).

^16^Count de Beaumont, General Stoytchev, Siperco, Reczek, Csanadi, Prince de Merode, Onesti, Kroutil.

^17^Fencing (Ferri), Weightlifting (State), Handball (Petit-Montgobert), Wrestling (Coulon), Canoeing (Coqueremont), Gymnastics (Gander), Judo (Ertel), Football (Chiazisoli Hockey (Glichitch), Cycling (Chesal), Skiing (Hodler), tir (Haßler), volleyball (Libaud).

^18^The last Olympic Congress was held in Berlin in 1930.

^19^DOSB: Deutscher Olympischer Sportbund (German Olympic Committee).

^20^Organización Deportiva Panamericana; Organización Deportiva Centroamericana y Caribe.

^21^The NOC of GDR did not attend the Munich GA and its absence was excused.

^22^Secretary general of the Belgian NOC.

^23^Secretary general of the British NOC.

^24^This committee was created to organise the Varna Congress and comprised representatives of the IOC, NOCs and Ifs. Studies and Projects Committee of the European NOCs: Brussels, 1974, 3rd Meeting, 10–14 January 1974, AENOC Collection, D-RM01-AACOE/040. Lausanne, Switzerland: IOC's Olympic Studies Centre.

^25^Voting result: Count de Beaumont 14 votes, Bengtson 10 votes, 3 blank votes.
